# *Symbiodinium* Community Composition in Scleractinian Corals Is Not Affected by Life-Long Exposure to Elevated Carbon Dioxide

**DOI:** 10.1371/journal.pone.0063985

**Published:** 2013-05-22

**Authors:** Sam H. C. Noonan, Katharina E. Fabricius, Craig Humphrey

**Affiliations:** Australian Institute of Marine Science, Townsville, Queensland, Australia; Leibniz Center for Tropical Marine Ecology, Germany

## Abstract

Ocean acidification (OA) is expected to negatively affect coral reefs, however little is known about how OA will change the coral-algal symbiosis on which reefs ultimately depend. This study investigated whether there would be differences in coral *Symbiodinium* types in response to OA, potentially improving coral performance. We used denaturing gradient gel electrophoresis (DGGE) of the internal transcribed spacer 2 (ITS2) region of ribosomal DNA to investigate the dominant types of *Symbiodinium* associating with six species of scleractinian coral that were exposed to elevated partial pressures of carbon dioxide (pCO_2_) *in situ* from settlement and throughout their lives. The study was conducted at three naturally occurring volcanic CO_2_ seeps (pCO_2_ ∼500 to 900 ppm, pH_Total_ 7.8 – 7.9) and adjacent control areas (pCO_2_ ∼390 ppm, pH_Total_ ∼8.0 – 8.05) in Papua New Guinea. The *Symbiodinium* associated with corals living in an extreme seep site (pCO_2_ >1000 ppm) were also examined. Ten clade C types and three clade D types dominated the 443 coral samples. *Symbiodinium* types strongly contrasted between coral species, however, no differences were observed due to CO_2_ exposure. Within five species, 85 – 95% of samples exhibited the same *Symbiodinium* type across all sites, with remaining rare types having no patterns attributable to CO_2_ exposure. The sixth species of coral displayed site specific differences in *Symbiodinium* types, unrelated to CO_2_ exposure. Symbiodinium types from the coral inhabiting the extreme CO_2_ seep site were found commonly throughout the moderate seeps and control areas. Our finding that symbiotic associations did not change in response to CO_2_ exposure suggest that, within the six coral hosts, none of the investigated 13 clade C and D *Symbiodinium* types had a selective advantage at high pCO_2_. Acclimatisation through changing symbiotic association therefore does not seem to be an option for Indo-Pacific corals to deal with future OA.

## Introduction

Present atmospheric carbon dioxide (CO_2_) levels have surpassed 390 ppm, the highest they have been in at least two million years [1]. Since the beginning of the industrial revolution, anthropogenic CO_2_ emissions, from the burning of fossil fuels and land clearing, have dramatically increased and continue to do so on a trajectory to reach or exceed 500ppm by the year 2100 [2].These increases are causing a planetary warming [3] and ocean acidification (OA). Following Henry’s gas law, as the partial pressure of atmospheric CO_2_ (pCO_2_) increases, more is dissolved into the surface waters of the world’s oceans, raising levels of dissolved inorganic carbon (DIC) and lowering carbonate saturation states and pH [4]. Declining carbonate saturation states are predicted to have negative consequences for calcifying organisms [5], however the increased levels of DIC may actually benefit some primary producers, enhancing the photosynthetic capacity of those limited by DIC [6]–[11].

Coral reefs are the most diverse marine ecosystems on our planet, primarily owing to the physical framework constructed by scleractinian corals as they secrete their calcium carbonate skeleton [12]. This process is made possible through a symbiotic relationship formed between the coral cnidarian host and single-celled photosynthetic dinoflagellates of the genus *Symbiodinium* [13]. Corals meet much of their energy requirements through translocation of photosynthetically fixed carbon from their symbionts [13]. While the coral host provides their *Symbiodinium* many of the substrates for photosynthesis, a significant proportion of the inorganic carbon required for fixation is still derived from the surrounding seawater [14]. Dinoflagellates, including *Symbiodinium,* are unique amongst eukaryotes in that they utilise type II ribulose biphosphate carboxylase/oxygenase (RuBisCO) during the onset of carbon fixation [15]. This enzyme has a much lower affinity with inorganic carbon than RuBisCo I [16], [17], leaving it under-saturated with CO_2_ under present-day pCO_2_ levels despite the apparent ability to also use bicarbonate (HCO_3_^−^) and the existence of a carbon concentrating mechanism (CCM) [18]. As pCO_2_ increases under OA, both CO_2_ and HCO_3_^−^ substrates for photosynthesis will become more abundant.

The genus *Symbiodinium* is presently delineated phylogenetically into nine lineages (clades A-I) using nuclear (18S, 28S, ITS1 and ITS2 regions) and chloroplast (23S) ribosomal DNA [19]–[21]. These clades are further divided into types which are usually identified by a single haploytype of the highly variable nuclear internal transcribed spacer (ITS1 and ITS2) regions of the rDNA operon [22]–[24]. While nuclear ribosomal DNA cistrons are multicopy regions, where there may be considerable intra-genomic variation among copies, they are frequently used to distinguish *Symbiodinium* types at ecologically relevant levels [23]–[28].

Different types of *Symbiodinium* are physiologically adapted to certain environments [25], [29], [30]. Indeed, *Symbiodinium* types may vary among geographic locations and along natural environmental gradients of temperature, depth and water quality [24], [26], [29], [31], [32], suggesting physiological differences [27], [33]. For example, both observational and experimental evidence indicates that *Symbiodinium* D types are generally more thermally tolerant than clade C types in the same coral host, and that a switch in dominance, from C to D, can occur in some hosts following heat stress [25], [27], [29], [34]. Recent work with *Symbiodinium in vitro* indicates that the physiological response to increased pCO_2_ may also be type specific; Brading *et al.* [10] showed that *in vitro* the growth and photosynthetic capacity of two different clade A *Symbiodinium* types increased with elevated pCO_2_, while that of another type A and a type B *Symbiodinium* remained unaffected. Types of *Symbiodinium* that are capable of utilising the more abundant pCO_2_ may therefore be expected to become dominant within a coral host and out-compete types that cannot [10], [35]. However, to date it remains unknown if corals are able to respond to rising CO_2_ concentrations by changing to better adapted dominant *Symbiodinium* types after long-term exposure to elevated pCO_2_ in the field.

Other studies that have investigated the response of *Symbiodinium* to OA were based on algal cultures [10], [36], relatively short-term exposure experiments of *in hospite Symbiodinium* communities in corals [9], [37]–[42] or *Symbiodinium* in host taxa other than corals [11], [43]. While these works have been informative, they do not have the capacity to predict the long-term effects of OA on potentially dynamic coral-algal symbioses.

Corals acquire their *Symbiodinium* either maternally, from already infected eggs or as brooded planula larvae (vertical transmission), or from the environment during the juvenile phase (horizontal transmission). Vertically transmitting species have been shown to have higher fidelity for *Symbiodinium* type than horizontal transmitters [24], [29]. Multiple *Symbiodinium* types can infect juveniles in horizontally transmitting coral species [33], [44]–[47] and recent work has identified multiple background symbiont types occurring within a single coral [48]–[50]. These features present avenues for symbiont differences to arise between con-specific colonies growing in different environments [51], [52].

The recent discovery of three volcanic CO_2_ seeps in Milne Bay, Papua New Guinea (PNG) [53], provides a unique opportunity to investigate the long term effects of increased pCO_2_ on the adjacent coral reef communities *in situ*. In this study we compare the dominant *Symbiodinium* types harboured by six species of scleractinian coral that have settled and grown within three CO_2_ seep sites to those of three adjacent control areas. The *Symbiodinium* type associating with a coral species from an extreme seep environment is also examined. Because the productivity of *Symbiodinium* may be limited by available inorganic carbon [16], [18], [36] and certain *Symbiodinium* types may be able to out compete others under OA scenarios [10], [43], we hypothesised that the frequency of certain *Symbiodinium* types within hosts may change at the seep sites in response to life-long exposure to high CO_2_.

## Materials and Methods

### Study Site and Species

Samples were collected from three shallow water (3–5m), CO_2_ seeps in Milne Bay, PNG, named Upa-Upasina, Esa’ Ala and Dobu. Three control areas with ambient pH conditions, one adjacent to each seep site, were also sampled. Samples were collected under research permit by the Department of Environment and Conservation of Papua New Guinea to the Australian Institute of Marine Science (AIMS). Seep and control sites are described in detail by Fabricius *et al.* [53]. Notably, seep areas are dominated by massive *Porites* spp. and scleractinian coral diversity declines sharply within the areas influenced by the seeps [53]. Sample collection at seep sites was restricted to areas with pH values of 7.8–7.9 (pCO_2_ ∼500 to 900 ppm) as this is what is predicted for the world’s oceans by the end of the century and thus considered ecologically relevant [2]. The extreme seep samples were collected from the seep at Upa-Upasina from the most intense bubbling areas where individual faviid and *Porites* coral colonies still occurred. Here pH values were observed to decline to a pH of 6.9 during the day (unpublished data), far beyond those predicted for the end of the century. Samples were collected on SCUBA over the course of three field trips from August 2010 to December 2011. During a sample collection dive, <2 cm coral fragments were removed from adult colonies of each species that were at least 5m apart, placed into separate bags, and preserved in 100% ethanol upon surfacing.

For the main CO_2_ comparison study, a total of 433 colonies were sampled from the six species of scleractinian coral across the six sites (three seep and three controls). The species sampled were *Acropora millepora, Pocillopora damicornis, Seriatopora hystrix, Porites cylindrica,* massive *Porites sp. and Galaxea fascicularis.* The massive *Porites sp.* designation potentially consisted of a number of *Porites* species with massive growth forms, and was left with the *sp.* label as accurate species identification was not obtained. A summary of the sample numbers for each species at each site is given in Table 1. The species of coral sampled included one horizontally transmitting species (*A. millepora*) and five vertically transmitting species. While it would have been preferable to sample more horizontally transmitting species it was not possible as there is an under-representation of these corals within the seep sites [53] and sufficient sample sizes were not attainable for other coral species at all sites. Two species are predominately brooders (*P. damicornis* and *S. hystrix*; both vertical transmitters) while the other species are broadcast spawners. Due to the calm conditions and absence of cyclones at Latitude 9°S and the <150 m length of the seep sites, there is little potential even for the branching colonies to have entered the seep sites via fragmentation rather than during settlement.

**Table 1:** The number of samples and the *Symbiodinium* ITS2 DGGE profiles for each coral species from each site used in this study.

Coral species^a^	Symb. Acqu.^b^	Dispersal^c^	b>Symb. Profile^d^		Upa-U Seep^e^	Upa-U Ctr	Dobu Seepb>	Dobu Crtb>	Esa’ A Seep	Esa’ A Ctr
*A. millepora*	Horizontal	Broadcast	Am1	15		15	10	13	13	12
			Am2	0		0	2	2	0	0
			Am3	0		0	2	0	0	3
*P. damicornis*	Vertical	Brooding	Pd1	15		15	15	14	14	13
			Pd2	0		0	0	1	1	1
*S. hystrix*	Vertical	Brooding	Sh1	15		14	0	8	0	0
			Sh2	0		1	0	0	0	0
			Sh3	0		0	0	2	9	14
			Sh4	0		0	0	3	0	1
			Sh5	0		0	15	2	6	0
*P. cylindrica*	Vertical	Broadcast	Pc1	7		10	0	9	10	10
			Pc2	1		0	1	0	0	0
			Pc3	1		0	0	0	0	0
*Porites sp.*	Vertical	Brooding	Pm1	10		10	10	9	8	10
			Pm2	0		0	0	1	1	0
			Pm3	0		0	0	0	1	0
*G. fascicularis*	Vertical	Broadcast	Gf1	2		3	1	1	2	0
			Gf2	8		8	9	5	9	10
*F. pentagona*	Horizontal	Broadcast	Fp1	2		0	0	0	0	0
			Fp2	8		0	0	0	0	0

a The coral species Acropora millepora, Pocillopora damicornis, Seriatopora hystrix, Porites cylindrica, massive Porites sp., Galaxea fascicularis and Favites pentagona used in this study. ^b^ The modes of Symbiodinium acquisition (Symb. Acqu.) employed by each coral species. Horizontal being from the environment (post-settlement) and vertical from maternal sources. ^c^The reproductive strategy of each coral species with either broadcast spawning gametes, larvae brooded in the parental colony or a combination of the two. ^d^The assigned Symbiodinium ITS2 DGGE profiles (Figures 1, 3). ^e^The Seep and control (Ctr) sites at Upa-Upasina (Upa-U), Dobu and Esa’ Ala (Esa’ A).

*Favites pentagona* was the only species that occurred in moderate abundance at the extreme seep site and 10 colonies were sampled from the Upa-Upasina seep, bringing the total sample number to 443 (Table 1). While this species was not used to compare *Symbiodinium* types between CO_2_ exposures and sites, it was investigated to examine whether extreme pCO_2_ environments would result in the occurrence of unusual *Symbiodinium* types.

### DNA extraction and Denaturing Gradient Gel Electrophoresis Profiling of the Internal Transcribed Spacer 2 region

DNA was extracted using a modified Chelex extraction protocol [54] which allows the inexpensive and rapid extraction of a high volume of samples. A small fragment of coral tissue of approximately 2 mm^2^ was removed from the coral branch with a fine pair of forceps and placed into a well of a 300 µL 96 well plate (Axygen). To each well 100 µL of extraction buffer was added. This buffer contained 10 µL of 20 g/L Proteinase K solution and 100 µL of 5% Chelex buffer (Chelex-100 BioRad) in 0.01 M Tris (pH 8.3). The plate was incubated at 55°C for 3 hours, with a vortex every hour, and then heat shocked at 95°C for 20 min to denature the Proteinase K enzyme. The plate was then centrifuged at 335.4 g for 5 min and stored at −20°C before polymerase chain reactions.

The use of denaturing gel gradient electrophoresis (DGGE) profiling of the internal transcribed spacer region 2 (ITS2) is a widely used method for identifying distinct *Symbiodinium* lineages [22], [23], [26], [55], [56] and was the method employed in this study. DGGE produces a profile for each sample that consists of one or more bands of the most numerically abundant ITS2 variants within the ribosomal array [28], that can differ from one another by a single base pair [22], [26], [55]. This results in multiple bands being evident in DGGE profiles. One µL of the supernatant from the Chelex extraction was taken as DNA template from each sample and amplified under standard conditions using the Multiplex Kit (Qiagen). The primers “ITS2 clamp” and “ITSintfor 2” were used in 12 µL reactions following a touchdown thermal cycle, including a 30 min final extension at 72°C, following LaJeunesse [57]. PCR products were visually checked on 1% agarose gels stained with ethidium bromide prior to DGGE. Amplified ITS2 PCR products were separated using 8% poly-acrylamide gels with a 35–55% denaturant gradient (formamide and urea) in an INGENY PhorU DGGE unit for 15 hrs at 75V. Gels were stained with SYBR Gold (Invitrogen) prior to examination on a transilluminator.

### Sequencing and Statistical analyses

Each sample was assigned a profile based on common banding patterns following Sampayo *et al*. [58]. Profiles are defined as the dominant subset of the *Symbiodinium* ITS2 community present within each sample. Profiles were assigned a *Symbiodinium* community by sequencing the dominant bands from at least two representative samples of each profile. In each case, identical sequences were obtained for the analogous bands in the same profile from different samples, confirming profile and band designations. These profiles were given an alphanumeric designation which comprised of a species code and then a profile number. For example Am1 and Am2 were two different profiles seen in *A. millepora*. Where samples from the same species were run on different gels the most common profiles from earlier gels were used as references on latter ones. A representative of each profile from each different gel was then run next to one another on a single gel to confirm category designations between gels. To determine symbiont type, a representative of each dominant band from the lowest relative position on the DGGE gel from each profile was cut from the gel, left to elute overnight in 40 µL of UV sterilized, ultrapure H_2_O, and re-amplified without the GC-rich reverse primer for direct sequencing in the forward direction (Macrogen Ltd., Korea). Following LaJeunesse *et al.* [26], bands that were relatively high on the DGGE gel were excluded from the study to minimise the sequencing of heteroduplexes that run higher on the gel as they denature more readily. Many of the minor and higher bands were also cut and sequenced to check for background types and/or heteroduplexes. However, these were excluded from later analyses as no patterns were evident between CO_2_ exposures and all minor bands clustered around the dominant band from the same profile. Each sequence was aligned with ClustalW and visually checked (BioEdit Sequence Alignment Editor) before being compared with sequences in the public library of GenBank (http://www.ncbi.nlm.nih.gov/BLAST/). An unrooted haplotype network was constructed from the sequence alignments using the program TCS (version 1.21). Networks were constructed by treating gaps as a fifth character state and with a 90% connection limit between haplotypes. Band II was included in the clade D network even though it had a 26 base pair indel as it matched the same Genbank sequences as the other clade D bands. Published sequences in Genbank that matched the newly obtained ITS2 sequences most closely were included in the haplotype networks for type identification.

A representative of each of the profiles, from all species, was then run on a single DGGE gel and, in conjunction with sequence data (to check for co-migration of dissimilar ITS2 types), the presence/absence of each of the dominant bands was scored to allow for between species comparisons of *Symbiodinium* profiles (Table S1) [58]. This presence/absence matrix was used to conduct a sequential permutational multivariate analysis of variance based on redundancy analysis to compare the distribution of *Symbiodinium* types between the six species, the three sites, and the two CO_2_ exposures nested within each of the three sites [59]. All statistics were completed using the vegan package in the statistical program R (version 2.15.1) [60].

## Results

A total of 20 *Symbiodinium* profiles, characterised by 13 distinct dominant bands in DGGE profiles, were identified across the seven species of coral (Figure 1). This includes both the six species of coral investigated in the main CO_2_ comparison study and *F. pentagona* from the extreme seep site. Twelve out of the 20 DGGE profiles contained more than one dominant band (Table S1), and often bands occurred in more than one profile within species. For example all but one of the profiles found in *P. cylindrica* and massive *Porites* sp. contained band IV, while band IX was common amongst three of the five *S. hystrix* profiles. Bands VI and VII displayed very similar migration across the DGGE gel (Figure 1), however sequence data indicated they differed by six base pairs (Figure 2). Four profiles were identical across coral species (Am1 and Pc3, Pm1 and Pc1, Pm3 and Pc2 as well as Gf2, Sh3 and Fp2) and the identity of these *Symbiodinium* communities was confirmed with the sequence data. All other profiles differed from one another by at least one dominant band. Therefore, the 443 samples contained a total of 15 distinct *Symbiodinium* communities.

**Figure 1:**
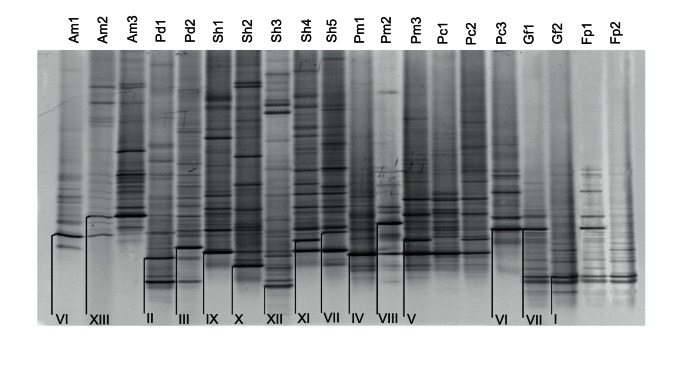
A representative of each *Symbiodinium* ITS2 DGGE profile from the seven coral species investigated. The *Symbiodinium* profiles from the species *Acropora millepora* (Am), *Pocillopora damicornis* (Pd), *Seriatopora hystrix* (Sh), *Porites cylindrica* (Pc), massive *Porites* sp. (Pm), *Galaxea fascicularis* (Gf) and *Favites pentagona* (Fp) are shown at the top of each DGGE column. Each of the 13 dominant bands (I-XIII), which characterise the profiles, are also indicated. Bands VI and VII are labelled twice as they appear to co-migrate, however sequence data differentiates them.

**Figure 2:**
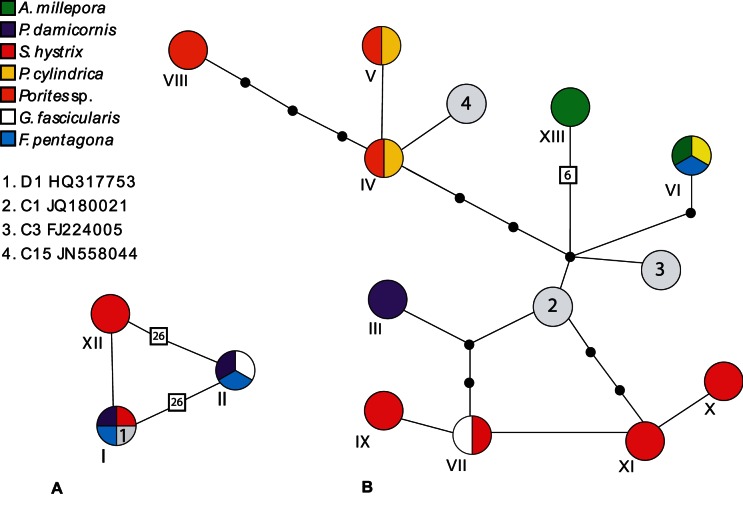
*Symbiodinium* ITS2 haplotype networks of the 13 dominant bands identified in this study. Parsimony networks of Clade D (A) and Clade C (B) *Symbiodinium* ITS2 haplotypes from dominant bands identified in this study. Coral species are shown in different colours and Roman numerals indicate dominant band numbers. Previously published sequences are also indicated (1–4) along with their Genbank accession number. Each node represents a base pair change and indel lengths are shown by the boxed numbers along branches. Bands III to XI and XIII represent clade C types, while bands I, II and XII represent clade D type *Symbiodinium.* Pies indicate the presence of *Symbiodinium* types in multiple coral species and are not indicative of frequency.

All 13 dominant ITS2 bands belonged to either clade C (61% of samples, 10 haplotypes) or D (39% of samples, 3 haplotypes). Twelve of these bands were novel types (Genbank accession numbers KC631398–KC631409), not previously recorded in the Genbank database. Some of these types differed from one another by a single base pair substitution or by a single insertion or deletion (Figure 2). While it was not the purpose of this study to name these new types, they did cluster most closely to D1 and C1, C3 and C15 (Genbank Accession numbers HQ317753, JQ180021, FJ224005 and JN558044, respectively) (Figure 2a and b). The ITS2 diversity in clade D was comparably low with band I matching the D1 sequence exactly, band XII being only one base pair different and band II being 26 base pairs different due to a large indel (Figure 2a). The clade C network was considerably more complex (Figure 2b). While none of the C type bands had a 100% match with the C1, C3 and C15 sequences, many clustered within a few base pair substitutions.

In the coral species *A. millepora,* all sequenced bands were closely related to C1 and C3 (Figure 2b). In *P. damicornis,* the abundant profile Pd1 contained ITS2 variants that either matched or clustered most closely to D1 while the less frequent profile, Pd2, was most closely related to C1. The *Symbiodinium* profiles of *S. hystrix* were the most diverse of the species investigated in the present study. Of these, the most common variants Sh1 as well as Sh5 and Sh3 clustered with both C1 and D1, respectively (Figure 2a and b). The majority of both the *P. cylindrica* and massive *Porites sp.* samples clustered with the C15 type, however one *P. cylindrica* sample was more closely related to C1 and C3. The vast majority of *G. fascicularis* samples contained profiles with bands that clustered with the D1 sequence only. The remaining *G. fascicularis* samples displayed the same banding pattern but also included an extra band that clustered closely with C1 (Figure 2a and b) indicating both *Symbiodinium* clades C and D were present within the same coral host.

**Figure 3:**
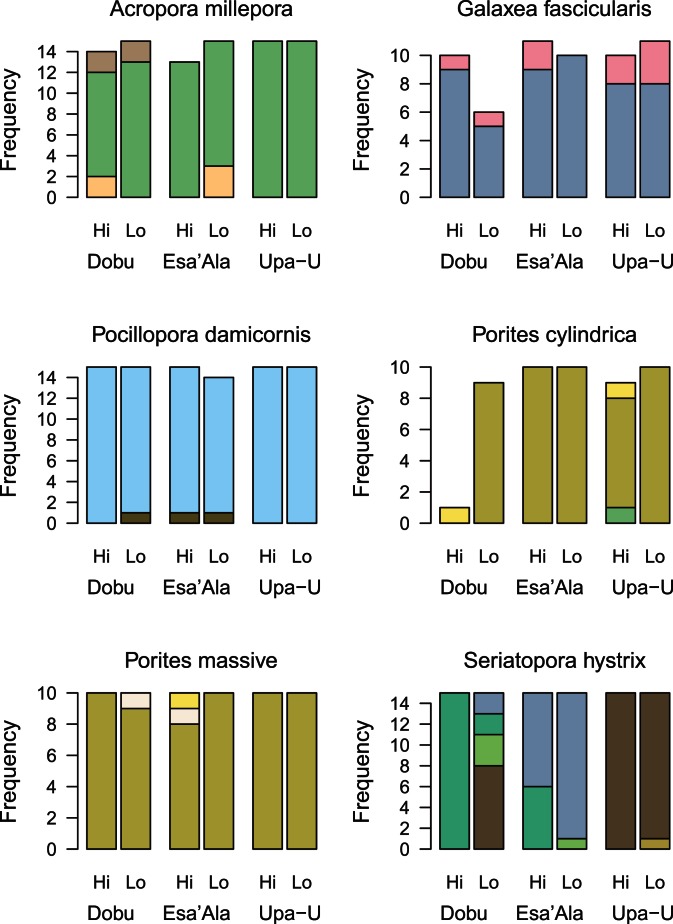
The frequency of different *Symbiodinium* ITS2 profiles between sites and CO_2_ exposures. The different *Symbiodinium* ITS2 profiles from the corals *Acropora millepora, Pocillopora damicornis, Seriatopora hystrix*, *Porites cylindrica*, massive *Porites sp.* and *Galaxea fascicularis* found at the three sites, each with High (Hi) and control (Lo) CO_2_ exposures. Each colour corresponds to one of the 14 unique *Symbiodinium* ITS2 profiles found in these coral species. Colours common between sites or coral species indicate synonymous *Symbiodinium* profiles (See also Table S1). The different colour schemes represent clade C (shades of green, yellow and brown), D (shade of grey and blue) and mixed (pink) *Symbiodinium.*

In the main CO_2_ comparison study differences between locations were minor in all of the six investigated species except *S. hystrix*, regardless of CO_2_ exposure (Figure 3). Approximately 85–95% of the samples exhibited the same symbiont profiles at all locations (Table 1). The remaining percentage comprised of rare types that only occurred in one or two samples, and for which no correlations were evident with CO_2_ exposures (Figure 3, Table 1). There were strong differences between species and weak differences between sites and CO_2_ exposures in *Symbiodinium* types when all coral species were combined in the one analysis (sequential permutation test for RDA, species: F_(5, 422)_ = 62.7, p = 0.01; site: F_(2, 422)_ = 4.5, p = 0.01; CO_2_ exposure nested within site: F_(3, 422)_ = 2.3, p = 0.01). This pattern was primarily driven by site-specific differences in *S. hystrix* profiles (sequential permutation test for RDA, site: F_(2, 84)_ = 27.6, p = 0.01; CO_2_ exposure nested within site: F_(3, 84)_ = 5.9, p = 0.01). For *S. hystrix,* the Dobu seep site was comprised entirely of Sh5, while Sh1 dominated both Upa-Upasina sites. The Esa’ Ala sites were dominated by Sh3, with one third of samples at the Esa’ Ala seep site characterised as Sh5 (Figure 3, Table 1). Profiles Sh1 and Sh5 were characterised by two dominant bands for which band IX was common between the two profiles (Figure 1). Band VII was also present in the Sh5 profile, differentiating it from Sh1, however it only deviated from band IX by a single base pair substitution (Figure 2). All other species were non-significant, however there was a marginally insignificant effect of CO_2_ exposure on *P. cylindrica* (sequential permutation test for RDA, site: F_(2, 43)_ = 1.0, p = 0.97; CO_2_ exposure nested within site: F_(3, 43)_ = 3.9, p = 0.06). which was not considered to be ecologically relevant (sampling was unbalanced as only one sample was found at Dobu High CO_2_, and only three of 49 samples yielded different types in the collection). As such, CO_2_ exposures did not lead to environmentally significant changes in symbiont types, regardless of the mode of symbiont acquisition or reproductive strategy, for all six coral species investigated in this study.

The groupings in the RDA plot based on the presence/absence of DGGE bands between profiles further indicated that some *Symbiodinium* types were common between coral species but that some coral species contained *Symbiodinium* types that differed from one another (Figure S1). This constrained analysis and the associated permutation analysis showed that differences between species were very strong compared to the differences between Sites and CO_2_ levels.

In the *F. pentagona* samples collected from the extreme seep site, all dominant *Symbiodinium* types were also observed in other coral species examined in this study. Eighty per cent of the *F. pentagona* samples had the Fp2 profile, consisting of bands closely related to D1, while the remaining samples (Fp1) also contained a clade C type (Band VI) (Figure 2). The profiles shared between *F. pentagona, G. fascicularis* and *S. hystrix* (Fp2, Gf2 and Sh4) as well as the joint occurrence of dominant bands between Fp1 and ten other profiles (Table S1) indicated that the *Symbiodinium* types that occur in the extreme seep area were also commonly found at both the less extreme seep and the control sites. As such, there was no evidence to suggest that the type of *Symbiodinium* associating with the seven species of coral investigated in this study was influenced by the exposure to the CO_2_ seeps.

## Discussion

This study shows that the dominant *Symbiodinium* community in scleractinian corals did not change despite a life-time (and for brooding species possibly even trans-generational) exposure to elevated concentrations of CO_2_ around volcanic CO_2_ seeps. The seep sites represent oceanic pCO_2_ conditions in line with IPCC scenarios predicted towards the year 2100, albeit without the predicted rise in temperature [2], [53]. While it was hypothesised that a change in *Symbiodinium* types would occur, no such change was observed. Instead, the *Symbiodinium* of five of the six coral species investigated between sites was dominated by a single ITS2 profile consisting of clade C or D types. The majority of symbiont types were consistent between sites within species, and some of the types were also observed in several coral species. Furthermore, the *Symbiodinium* types found in a seventh species of coral from the extreme seep area (dominated by types similar to D1) were also found commonly at the moderate seep and control areas.

The *Symbiodinium* types identified in the present study clustered closely to C1, C3, C15 and D1 sequences from Genbank, however the vast majority were novel types whose ITS2 haplotype had not previously been recorded. *Symbiodinium* types C1, C3, C15 and D1 are common throughout the Indo-Pacific and may form symbiosis with a variety of taxa [26]. To date there have been hundreds of unique *Symbiodinium* ITS2 haplotypes reported [57] and, as per the present study, new sites often reveal further diversity [56]. The ITS2 diversity of clade C *Symbiodinium* types is greater than that of clade D types [31], [56], [61], [62]. The high representation of D type *Symbiodinium* in the present study (found in 4 of the seven species, at a total of 39% of all samples) may reflect the low latitude and subsequent warm waters of the study sites (approximately 9° South), as the frequency of certain D1 types have been observed to increase in warm waters [25], [29], [30].

The few studies that have investigated the physiological response of *Symbiodinium* to OA, either *in hospite* or in culture, have found conflicting results. Increased DIC and pCO_2_ has been reported to increase net production in some studies [10], [11], [39], [40], [43], while others have found negligible or negative effects [4], [36]–[38]. These studies not only utilised different experimental methodologies and host species, but few have identified the sub-cladal type of *Symbiodinium* under experimentation, further limiting comparisons. Work by Brading *et al.* [10] indicated that in culture, two A type *Symbiodinium* were better able to take advantage of elevated levels of inorganic carbon than another type A and type B through increased growth and photosynthesis. Clade C and D types, which are dominant in corals of the Indo-Pacific [26], [30], [63], have not been subject to similar physiological studies, and such work on common Indo-Pacific Clade C and D type *Symbiodinium* is warranted.

In the present study there was no indication that the coral investigated had acclimatised to high pCO_2_ at the seeps by changing their dominant type of *Symbiodinium*. If indeed certain *Symbiodinium* types outperform others in response to OA [35], those types were not found at the study sites due to environmental or geographic constraints [23], [26], [29], or in the host species investigated due to host-symbiont specificity [33], [64]–[66]. While more work at CO_2_ seep sites is needed to determine if the increased DIC and pCO_2_ increases production in coral holobionts *in situ*, we have found no evidence to suggest that any difference is sufficient enough for one *Symbiodinium* type to outcompete another. Moreover, recent work by Howells *et al.* [67] indicates that there may be substantial adaptation within the same sub-cladal types of *Symbiodinium* to local environmental conditions. This indicates that there is potential for the seep *Symbiodinium* to have undergone local adaptation to the OA conditions that is sufficient to prevent selection of certain types over others. Physiological studies that monitor the response of both the coral and the algal partners, as well as fine scale population genetic studies, are needed to identify any potential local acclimatisation or even adaptations.

The diversity of coral communities is sharply reduced at the three seep sites compared with the control sites, although coral cover remains similar [53]. Seep communities are dominated by massive *Porites* spp., while adjacent control reefs are comparatively rich in *Acropora* spp. [53]. Our study has shown that the massive *Porites* sp. at the seeps house the same C15-like *Symbiodinium* as at the control sites. It is possible that C15-like types can take advantage of the additional CO_2_, buffering the host from the negative effects of OA. However, it is unlikely that the association with C15-like *Symbiodinium* types alone accounts for the dominance of massive *Porites* spp. at seep sites, as *P. cylindrica* contained the same C15-like *Symbiodinium* but is uncommon at the seeps [53]. It is hypothesised that the observed difference in community structure may therefore be related to differences in the inherent stress tolerances of the coral hosts themselves [34], resulting in shifts in competitive advantages from sensitive to persistent and long-lived taxa. Massive *Porites* are comparatively tolerant to a variety of stressors [68]–[71] and may be less affected by the negative effects of OA compared to branching *Acropora* spp. [38], [72], [73].

Reduced recruitment success at high pCO_2_ may also contribute to the observed shift in coral community structure. Of the coral species examined in the present study, *A. millepora* was the sole horizontally transmitting species that occurred at sufficient numbers to be sampled at seep sites. Such under-representation of horizontally transmitting species at the seeps may be due to constraints intrinsic to their mode of symbiont acquisition, potentially suggesting a high sensitivity of free-living *Symbiodinium* to high CO_2_. This theory appears possible as about 75% of Pacific coral species are horizontally transmitting [74], yet very few are found at the seep sites. Although juvenile *A. millepora* are obviously able to take up symbionts at the seep sites, even moderate declines in algal infection rates under OA may reduce recruitment success of horizontal transmitters [42], potentially contributing to their under-representation in the coral community.

This study has shown that the observed differences in scleractinian coral communities at the Milne Bay CO_2_ seep sites is unlikely to be due to differences in the dominant type of *Symbiodinium* harboured by the particularly successful corals. The data suggest that the inherent stress tolerance and resilience of the coral holobiont, rather than a change in symbiotic association with more tolerant *Symbiodinium* types, determined the ability of massive *Porites* to live under high CO_2_ conditions. No evidence was detected to suggest that any of the other coral species may be able to adapt or acclimatise to OA conditions by switching or shuffling the dominant type of *Symbiodinium* they harbour to types that are better able to utilise the more abundant DIC. This was reiterated by the overlap in types found commonly throughout control sites and the extreme seep site. However, the relative contribution of reduced recruitment success of horizontally transmitting corals, and the physiological performance of sensitive host corals associated with different *Symbiodinium* types requires further investigation to better understand the underlying mechanisms responsible for structuring these coral reef communities, and to predict how coral reefs will be shaped by ongoing and rapidly progressing acidification of the world’s oceans.

## Supporting Information

Figure S1 **Redundancy analysis plot of the similarity of banding patterns across the 443 coral samples.** Each point signifies a unique combination of bands (14 in total), corresponding to the profiles observed in the corals *Acropora millepora* (Am), *Pocillopora damicornis* (Pd), *Seriatopora hystrix* (Sh), *Porites cylindrica* (Pc), massive *Porites* sp. (Pm), *Galaxea fascicularis* (Gf) and *Favites pentagona* (Fp) from the main CO_2_ comparison study. The arrows point towards the highest representations of each coral species (red arrows), sites with high (hi) and low (lo) CO_2_ exposure (blue arrows), with the length of the arrows signifying strength of association. Sequential permutation test for RDA, for species: F_(5, 422)_ = 62.7, p = 0.01; site: F_(2, 422)_ = 4.5, p = 0.01; CO_2_ exposure nested within site: F_(3, 422)_ = 2.3, p = 0.01).
(EPS)

Table S1 The presence-absence matrix of *Symbiodinium* ITS2 bands between the profiles observed in *Acropora millepora* (Am), *Pocillopora damicornis* (Pd), *Seriatopora hystrix* (Sh), *Porites cylindrica* (Pc), massive Porites sp. (Pm), *Galaxea fascicularis* (Gf) and *Favites pentagona* (Fp). Bands III to XI and XIII represent clade C types, while bands I, II and XII represent clade D type *Symbiodinium*.
(DOCX)
